# Analysis of the Spatiotemporal Digestion Characteristics of Pine Pollen Processed by Different Methods in Middle-Aged Adults Using an In Vitro Digestion Model System

**DOI:** 10.3390/foods15111887

**Published:** 2026-05-27

**Authors:** Kexin Yu, Danyang Liang, Xinlei Yang, Xixian Lv, Yin Yin, Yuqin Wang, Minjie Gao, Zhitao Li, Yan Yan

**Affiliations:** 1 School of Grain Science and Technology, Jiangsu University of Science and Technology, Zhenjiang 212100, China; 2 State Key Laboratory of Food Science and Technology, Jiangnan University, Wuxi 214122, China; 3 Key Laboratory of Carbohydrate Chemistry and Biotechnology, Ministry of Education, School of Biotechnology, Jiangnan University, Wuxi 214122, China; 4 Guizhou Key Laboratory of New Quality Processing and Storage of Ecological Specialty Food, Guiyang 550025, China; 5 School of Liquor and Food Engineering, Guizhou University, Guiyang 550025, China

**Keywords:** pine pollen, processing methods, middle-aged people, in vitro dynamic bionic digestion, gut microbiota, colonic fermentation

## Abstract

This study utilized a proprietary dynamic biomimetic digestion reactor to compare the differential behaviors of broken-wall pine pollen (PB), whole-wall pine pollen (WPB), and pine pollen wall extract (T) during simulated gastrointestinal digestion and colonic fermentation in middle-aged individuals. Morphological changes were observed using scanning electron microscopy, and glucose release, enzyme activity, intestinal gas composition, and gut microbiota structure were dynamically monitored. The results indicate that cell wall disruption significantly accelerated structural breakdown, resulting in the highest glucose release, superoxide dismutase, and lipase activities during the gastric and small intestinal phases, as well as the highest activity of alkaline phosphatase and H_2_ and CO_2_ gases during colonic fermentation. Due to its intact outer wall, WPB exhibited more robust and sustained enzyme activity and gas production, which was particularly beneficial for maintaining catalase activity in the descending colon of women. The T group demonstrated exceptional glucose and flavonoid release during digestion, but exhibited low SOD activity in the colon and a specific increase in H_2_S and VOCs in the descending colon. Furthermore, all three groups inhibited *Escherichia-Shigella*, with gender differences observed in the regulatory patterns. This study elucidates the processing-driven differential regulatory characteristics of pine pollen on in vitro intestinal fermentation behaviors, providing an in vitro experimental basis for the development of differentiated pine pollen products tailored to the needs of different populations.

## 1. Introduction

Pine pollen consists of the male spores of pine species, such as the Chinese red pine and the Chinese white pine [1], and possesses significant nutritional and medicinal value. China is the largest producer of pine pollen, with a theoretical annual natural reserve exceeding 30 million tons. However, the current actual commercial industrial output of edible pine pollen and its derived products in China is only several thousand tons, indicating a substantial untapped potential and developmental waste of this natural wild plant resource [2]. Pine pollen is rich in various bioactive compounds beneficial to human health, including proteins [3], vitamins, unsaturated fatty acids [4], polyphenolic compounds [5] and carbohydrates [6]. In addition, pine pollen contains various enzymes and coenzymes that play important roles in human digestion, absorption, and cellular metabolism, such as superoxide dismutase, lipase, and catalase [2]. Pine pollen offers a variety of health benefits, including promoting wound healing [7], inhibiting tumor growth [8], providing antioxidant effects [9], regulating metabolism [10], reducing inflammation, and improving gastrointestinal function [11]. Based on processing methods, pine pollen can be classified into three categories: cell-wall-broken pine pollen, unbroken-cell-wall pine pollen, and pine pollen wall extract [7,12]. The effects of consuming pine pollen in different forms vary to some extent. When the cell walls of pine pollen are broken down, polysaccharides, flavonoids, and fat-soluble components are released and absorbed more easily, which typically leads to a significant increase in antioxidant activity and bioavailability. This helps promote changes in enzyme activity, such as the utilization of antioxidant enzymes and digestive enzymes [12,13]. In contrast, unbroken pine pollen retains its intact outer wall, and many of its active ingredients are restricted by the structure of the spore powder, resulting in lower absorption rates. However, the pine pollen wall extract is mainly enriched with indigestible wall components, including sporopollenin, cellulose, hemicellulose, and lignin-like polymers. In addition, the extract retains a portion of resistant polysaccharides, lipid trace elements, and small amounts of wall-associated proteins that are tightly bound to the cell wall. Some of these components may serve as prebiotic carriers or influence the production of short-chain fatty acids, thereby modulating the composition and metabolism of the gut microbiota [14,15].

The gut microbiota is closely linked to human health and disease [16]. Middle-aged adults often face declining health and reduced life expectancy [17], and their gut microbiota undergoes changes and is influenced by dietary protein intake. The abundant nutrients in pine pollen can effectively support and regulate the body’s normal functions and metabolism [2]. Therefore, a systematic study of the extent to which pine pollen is digested and utilized in the digestive tract, as well as its regulatory effects on the gut microbiota and metabolism, is crucial for assessing its potential as a functional food. Most current studies on pine pollen are based on animal experiments. For example, Wang et al. found through mouse experiments that pine pollen and its polysaccharides can influence the levels of inflammatory factors and the structure of the microbiome [18]. However, existing research indicates that there are fundamental differences between the gut microbiota of laboratory mice and humans, and mouse models have limitations in fully replicating the human digestive system [19]. As a result, in vitro digestion models have garnered significant attention as an efficient alternative tool; these models are categorized into static and dynamic types. Due to their low cost and ease of use, static models are often employed in preliminary laboratory research [20]. However, they cannot simulate the physical processes of gastric emptying and intestinal peristalsis in the human body [21], and they have limitations in simulating the intestinal colonization process and maintaining a strictly anaerobic environment. Compared to static models, dynamic models can simulate dynamic pH changes, continuous secretion of digestive fluids, and physical processes such as shearing and grinding, thereby more accurately replicating the physiological environment of the gastrointestinal tract. Although multi-compartment dynamic models such as SHIME^®^ have proven their value in assessing the effects of food additives on the gut microbiota [22], they still fall short in simulating biomimetic peristalsis [23]. The BGR-7 Spatio-Temporal Dynamic Evaluation System for the Gastrointestinal Tract, independently developed by our laboratory, is capable of simulating the motility characteristics of the human gastrointestinal tract while simultaneously monitoring real-time changes in pH and gas concentration [24]. To date, this reactor has been successfully applied in numerous studies of functional ingredients; for example, Zhou et al. used this model to investigate the spatiotemporal digestion and gut microbial fermentation characteristics of Ganoderma lucidum [25], while Liu et al. employed it to analyze the potential prebiotic effects of blackberry seed polysaccharides [26].

The current consumer market offers a wide variety of pine pollen products, but the mechanisms by which these products regulate gut health in middle-aged adults remain unclear. Therefore, in this study, we aim to investigate the release of glucose following gastrointestinal digestion of different types of pine pollen, as well as changes in the activity of enzymes such as superoxide dismutase, α-amylase, and lipase. We further examined their effects on gut microbiota composition and intestinal gas production, as well as their interactive effects. This study aims to elucidate the outcomes of digesting different types of pine pollen, determine which type is more beneficial for gut health in middle-aged adults, and lay the groundwork for further research in this field.

## 2. Materials and Methods

### 2.1. Materials

Guozhen brand broken-wall pine pollen (PB, with a wall-broken rate of at least 99%) and whole-wall pine pollen (WPB) are supplied by New Era Health Industry (Group) Co., Ltd. (Beijing, China). The Guozhen brand broken pine pollen used in this study was provided by New Era Health Industry (Group) Co., Ltd. (Beijing, China), which employs advanced ultra-low temperature high-speed airflow crushing technology. According to the quality control standards provided by the manufacturer, the uniformity of the wall-broken rate of this batch of product was strictly monitored, and the wall-broken rate was ≥99%. Pine pollen broken-wall extract (T) was extracted from broken pine pollen according to the invention patent titled ‘A Method for Extracting Pine Pollen Wall from Broken Pine Pollen’ (Patent No. CN 111671069 A). All pine pollen samples used in this experiment were derived from Pinus massoniana and Pinus tabuliformis. Pectin, xylan, arabinogalactan, mucinyeast extract, trypticase, casein, L-cysteine, MgSO_4_·7H_2_O, FeSO_4_·7H2O, CaCl_2_·6H_2_O, KCl, NaCl, K_2_HPO_4_, KH_2_PO_4_, hemin, sodium cholic acid, and other reagents were purchased from China McLean and Aladdin Reagent Company (Shanghai, China).

### 2.2. Sources and Collection of Fecal Samples

Fresh fecal samples were obtained from six middle-aged men and six middle-aged women (all aged 40–59 years) [27]. All donors must maintain a regular diet and sleep schedule, be free of serious illnesses—particularly those that affect the gut microbiota—and must not have taken any probiotics or prebiotics in the two months prior to donation. Collect fresh fecal samples into sterile vials, then mix and dilute the samples with sterile phosphate-buffered saline (pH 7.3) to prepare a 10% fecal suspension (*w*/*v*). Thoroughly mix the suspension and filter it through four layers of sterile gauze to remove food particles. To minimize the impact of inter-donor variability, fecal samples from six volunteers were mixed in equal proportions to produce the final fecal suspension. To preserve the sex-specific characteristics of the gut microbiota, samples from males and females were separately and independently pooled. Subsequent in vitro colonic fermentation experiments were conducted independently using the male pooled slurry and the female pooled slurry. All volunteers provided written informed consent (Approval No. S2021-10-008).

### 2.3. In Vitro Simulation of Gastrointestinal Digestion

Simulated saliva and simulated gastric juice were prepared by referring to the relevant literature and making minor modifications [28]. The simulated saliva consists of KSCN (0.5816 g), NaCl (5.0996 g), KCl (2.6064 g), NaHCO_3_ (2.4640 g), Na_2_SO_4_ (1.6580 g), NaH_2_PO_4_ (2.5832 g), urea (0.7272 g), α-amylase (170 U/mL), and 40 mL of distilled water. The simulated gastric electrolyte solution was prepared by dissolving NaCl (620.0 mg), KCl (220.0 mg), CaCl_2_ (30.0 mg), and NaHCO_3_ (120.0 mg) in 200.0 mL of distilled water, and the pH was adjusted to 2.0 using 0.1 mol HCl. Take 40 mL of gastric electrolyte solution, then add 3.2 U/mL pepsin, 20 U/mL gastric lipase, and 1.0 mol/L CH_3_COONa solution to prepare simulated gastric juice. Following the method described in [29,30] for simulating intestinal fluid, first mix NaCl (5.4 g), KCl (650.0 mg), CaCl_2_ (330.0 mg), and 200 mL of distilled water until thoroughly blended. The small intestine electrolyte solution was then prepared by adjusting the pH to 6.3 using NaHCO_3_. Add 13.0 mg of trypsin and 5.0 g of 4% bile salt solution (*w*/*w*) to 100.0 g of small intestine electrolyte solution. Adjust the pH of the duodenal, jejunal, and ileal fluids to 6, 6.5, and 7.0, respectively.

In vitro simulation of saliva-gastrointestinal digestion was performed based on previously reported methods, with minor modifications [31,32]. First, 6 g of pine pollen was mixed with artificial saliva in a 1:1 (g:mL) ratio [20], and the mixture was incubated for 10 min at 37 °C under magnetic stirring at 150 rpm to simulate oral digestion. Next, the oral digestive fluid was mixed with simulated gastric fluid in a 1:1 (mL:mL) ratio, the mixture was transferred to the simulated gastric reactor of the BGR-7 system, and was digested for 2 h at 37 °C under simulated peristaltic conditions (approximately 3 rad/min). Once digestion in the stomach was completed, the digestive fluid was pumped into the simulated small intestine reactor at a flow rate of 1 mL/minute. Digestion in the intestines was performed in three consecutive stages: the duodenum, the jejunum, and the ileum. At each stage, the digestive fluid was mixed with the corresponding simulated intestinal fluid in a 1:1 ratio, and the simulated peristaltic frequencies for the duodenum, jejunum, and ileum were set to 6, 7, and 8 rad/min, respectively. At 37 °C, each stage lasted for 1 h. Digestate samples were collected aseptically at the end of the gastric phase and at the end of each intestinal phase.

### 2.4. In Vitro Simulation of Colonic Fermentation

The BGR-7 biomimetic colon bioreactor system consists of three consecutive fermentation units, which simulate the proximal ascending colon (pH = 6.5), the transverse colon (pH = 7.0), and the distal descending colon (pH = 7.5), respectively. 250 mL of sterile basal medium (BHI 38.5 g/L, mucin 1 g/L, Na_2_SO_4_ 3 g/L) was added to each reactor and was purged three times with nitrogen to establish an anaerobic environment. Once the reaction system stabilized, it was inoculated with the pretreated fecal homogenate at a ratio of 8% (mL:mL). After sealing, the system temperature is maintained at 37–37.5 °C, with a simulated peristaltic frequency of 2–4 rad/min. To simulate a continuous supply of nutrients, the basal medium was replenished at a constant rate of 120 mL/day. Throughout the fermentation process, fermentation broth samples were collected from the sampling port every 4 h using a sterile syringe, placed immediately on ice, and then transferred to −80 °C for storage for subsequent analysis.

### 2.5. Scanning Electron Microscopy (SEM) Analysis

Using a scanning electron microscope, images of crushed and uncrushed pine pollen during the digestion process were captured at different magnifications to observe changes in their internal (cross-sectional) and external morphology. The samples collected during digestion in the stomach, small intestine, and colon were centrifuged at 4000 rpm for 10 min. Subsequently, the pellets were freeze-dried and sputter-coated with platinum to enhance conductivity and prevent sample charging during testing. A small amount of the prepared sample powder was adhered to conductive tape and fixed onto the SEM sample stage. The surface morphology of the samples was imaged using a Hitachi High-Technologies field-emission scanning electron microscope (Hitachi, SU8200, Tokyo, Japan) at an accelerating voltage of 5 kV. Images were captured at a working distance of 8–10 mm, with magnifications of 800× and 5000×.

### 2.6. Determination of Glucose Content

The glucose content released during digestion and fermentation was quantitatively determined using an SBA-40E biosensor analyzer (Shandong Academy of Sciences Institute of Biology, Shandong, China). As an industry standard method for offline detection, this analyzer is based on the specific catalytic reaction of an immobilized glucose oxidase sensor at its core [33]. The specific operation procedure followed the standard biosensing analytical protocol: after the system had stabilized, 25 μL of a glucose standard sample was first aspirated for two-point system calibration. After calibration was completed and the system RSD met the required criteria, 25 μL of the in vitro digestive fluid or fermentation broth sample was directly injected. The glucose concentration (mg/100 mL) in the sample was then recorded and output in real time by a microcomputer-based intelligent system, which was used to evaluate the carbohydrate hydrolysis and substrate utilization kinetics of pine pollen subjected to different processing methods at various stages.

### 2.7. Determination of Enzyme Activity and Related Parameters

The superoxide dismutase activity was determined using the Solarbio Superoxide Dismutase Activity Assay Kit. Alpha-amylase activity was determined using Boxbio’s Alpha-Amylase (α-AL) Activity Assay Kit. Lipase activity was determined using Solarbio’s Lipase Activity Assay Kit. Plant flavonoid content was determined using Boxbio’s Plant Flavonoid Content Assay Kit. The sample solution was prepared according to the BoxBio kit instructions. After being allowed to stand at room temperature for 5 min, 200 μL of the reaction mixture was pipetted into a 96-well plate. The absorbance values for each group at a wavelength of 415 nm were measured and recorded using a microplate reader. Using the measured absorbance values and the concentration gradient of the standard samples, a standard curve is plotted to determine the catalase activity in the sample. The sample solution was prepared according to the instructions for the BoxBio kit. 200 μL of the sample solution was pipetted into a 96-well plate, the absorbance at 510 nm was measured, and the alkaline phosphatase activity in the sample was determined.

### 2.8. Analysis of Gas Composition and Concentrations

A BGR gas detection system was used to collect and analyze intestinal gas production. Use electrochemical gas detectors to monitor the concentrations of CO_2_, H_2_, H_2_S, and VOCs in the colon reactor in real time. The formula for calculating intestinal gas volume is as follows:

Gas content(ppm) = (V1/V2) × n

V1: Volume of the gas detection device; V2: Volume of gas collected during fermentation in the biomimetic colon reactor; n: Data displayed by the gas detection device.

### 2.9. 16S rRNA Sequencing Analysis

After completing genomic DNA extraction, PCR-targeted amplification was performed on the V3 + V4 regions of 16S rDNA. Sequencing adapters were ligated to the purified amplicons, a sequencing library was constructed, and sequencing was run on an Illumina platform. After raw reads were obtained from sequencing, the DADA2 software (version 1.14.1) was used to filter and correct the reads, and the non-redundant reads along with their corresponding abundance information were output. Then, the reads were assembled into tags, and chimeric tags were removed to obtain the tag sequences and abundances for subsequent analysis—that is, ASV sequences and ASV abundance information. Alpha diversity and principal coordinate analysis (PCoA) were used to detect differences within groups, while the Adonis test was used to detect differences in microbial community structure.

### 2.10. Statistical Analysis

All experiments were conducted in three independent replicates, and data are presented as mean ± standard deviation. We used SPSS 27.0 software to perform one-way analysis of variance (ANOVA) and Tukey’s post hoc test to assess the significance of differences between groups. Plots were generated using Origin 2025 software, with *p* < 0.05 set as the threshold for statistical significance.

## 3. Results

### 3.1. Morphological Changes During the Digestion of Pine Pollen

**Figure 1:**
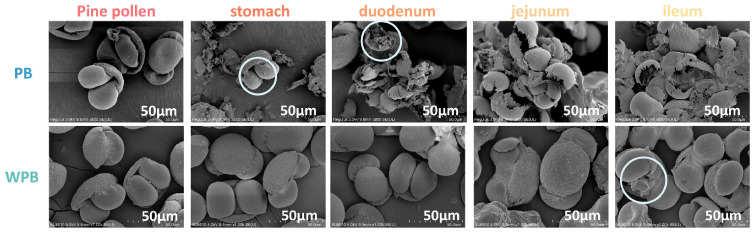
Scanning electron microscope images of broken-wall pine pollen (PB) and unbroken-wall pine pollen (WPB) during digestion in the stomach and small intestine.

Figure 1 presents scanning electron microscopy (SEM) images of broken pine pollen (PB) and unbroken pine pollen (WPB) after simulated gastric and small intestinal digestion. Pine pollen consists of a central chamber containing sporoplasm and two hollow air sacs. At 800× magnification, PB exhibited partial rupture of the shell surface after 2 h of gastric digestion. After 1 h of duodenal digestion, extensive shell rupture occurred, revealing a porous inner layer or fragmented structure with a honeycomb-like fracture surface. Following 1 h of jejunal digestion, some particles showed collapse, and the internal honeycomb-like structure was partially digested. During the ileal stage, more pronounced fragmentation was observed, with severe particle disintegration and extensive exposure of internal contents. In contrast, whole-wall pine pollen (WPB) exhibited extremely high structural stability. At 1000× magnification, no obvious rupture was observed in WPB after gastric, duodenal, or jejunal digestion. After ileal digestion, only a few particles displayed cracks, while the overall structure remained largely unchanged, and no significant leakage of internal contents was detected.

This confirms the extraordinary resistance of the sporopollenin exine to digestive degradation [34]; however, its high stability also limits the release of internal substances [35]. In contrast, wall-broken treatment significantly increases the probability of structural disintegration and content release at various stages of the digestive tract. Therefore, this may enhance the contact of nutrients (such as polysaccharides, proteins, and flavonoids) with digestive enzymes or solvents, thereby improving their bioavailability [36] and making them more readily available for microbial utilization during fermentation [37].

### 3.2. Changes in Glucose Content During Gastric and Intestinal Digestion and Colonic Fermentation of Pine Pollen

**Figure 2:**
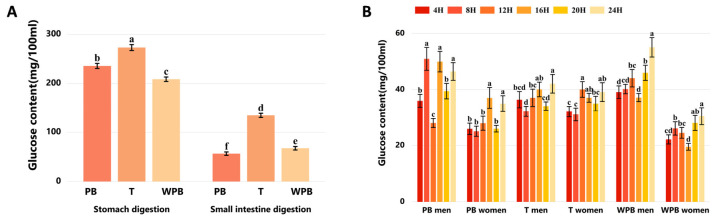
(**A**): Glucose levels during digestion in the stomach and small intestine; (**B**): Glucose levels during colonic fermentation. Error bars represent the standard deviation of three independent experiments. Means with different letters are significantly different (*p* < 0.05).

The key bioactive components of pine pollen include a range of pharmacological activities: antitumor, hepatoprotective, lipid-lowering, anti-inflammatory, immunomodulatory, antioxidant, antiviral, and antibacterial properties [38]. Since the carbohydrates in pine pollen are gradually degraded during digestion and release glucose, monitoring the level of glucose released at each digestive stage serves as a key indicator for evaluating the effects of different processing methods on the nutrient release of pine pollen. Figure 2A illustrates the specific release of glucose across different treatment groups during simulated gastrointestinal digestion.

After digestion in the stomach, Sample T had the highest glucose concentration, reaching 273.0 mg/100 mL. This may be because the pretreatment released free glucose or easily degradable small-molecule sugars. The glucose content of PB (236.5 mg/100 mL) is slightly higher than that of WPB (208.5 mg/100 mL), and this difference suggests that breaking the pine pollen wall facilitates the release of glucose contained in the pollen [13,39]. However, high release rates in the stomach do not necessarily mean that the substance can be efficiently absorbed by the body. The final absorption of glucose and other soluble components also depends on the efficiency of their enzymatic digestion in the small intestine and microbial fermentation in the colon. During the glucose release phase in the small intestine, the T sample still exhibited the highest glucose concentration (134.25 mg/100 mL), significantly higher than that of the PB and WPB samples. This further confirms the excellent results achieved by using technological methods to completely break through the external barrier of pine pollen and promote glucose release.

As shown in Figure 2B, in the colonic phase, male subjects in the PB group exhibited significant fluctuations, with a peak of 51.0 mg/100 mL at 8 h, a low of 28.0 mg/100 mL at 12 h, and 46.5 mg/100 mL at 24 h. This may indicate that although the polysaccharides in the broken-wall pine pollen can be rapidly fermented by the colonic microbiota to release glucose, there is a lag in the microbiota’s utilization of glucose, leading to a dynamic process characterized by initial accumulation, mid-phase consumption, and subsequent re-release through secondary fermentation in the late phase. In contrast, the figures for women were generally more stable but lower. There were no significant differences in gender within Group T; the 24 h levels rose to 42.1 mg/100 mL in males and 39.1 mg/100 mL in females, which may suggest that the broken-wall pine pollen extract has a balanced effect on promoting metabolism. Glucose levels in the WPB group continued to rise, reaching 55 mg/100 mL after 24 h, indicating that glucose release exceeded utilization. Glucose levels in women after fermentation were significantly lower than those in men, at only 30.5 mg/100 mL after 24 h. Overall, middle-aged women exhibited superior metabolic efficiency in the colon, suggesting that their microbial activity may be more advantageous than that of men.

### 3.3. Changes in Enzyme Activity During the Gastric and Intestinal Digestion and Colonic Fermentation of Pine Pollen

Pine pollen also contains a variety of enzymes and coenzymes that have positive effects on human digestion and absorption, such as superoxide dismutase, α-amylase, and peroxidase [2,40]. Therefore, measuring the release of these enzymes during digestion is essential for studying the degree of pine pollen digestion.

**Figure 3:**
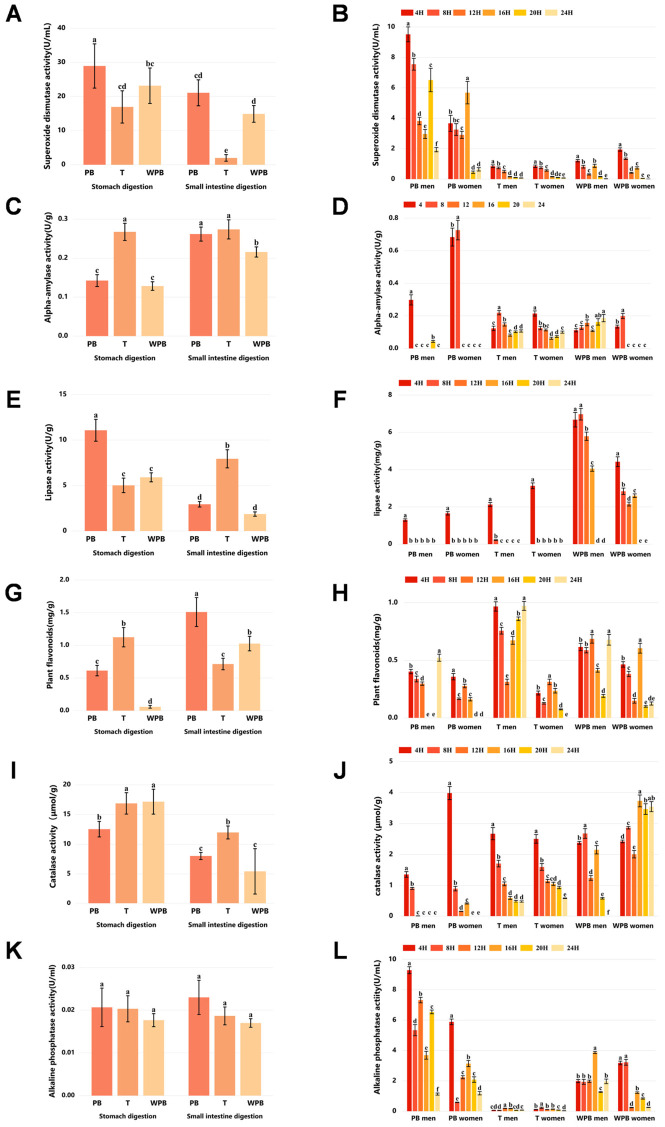
Changes in enzyme activity in three samples following in vitro simulation of gastric and small intestinal digestion and colonic fermentation; (**A**): Superoxide dismutase activity after gastric and small intestinal digestion; (**B**): Changes in superoxide dismutase activity during colonic fermentation; (**C**): α-amylase activity after gastric and small intestinal digestion; (**D**): Changes in α-amylase activity during colonic fermentation; (**E**): Lipase activity after gastrointestinal digestion; (**F**): Changes in lipase activity during colonic fermentation; (**G**): Flavonoid content after gastrointestinal digestion; (**H**): Changes in the content of flavonoids during colonic fermentation; (**I**): Catalase activity after gastrointestinal digestion; (**J**): Changes in catalase activity during colonic fermentation; (**K**): Alkaline phosphatase activity after gastrointestinal digestion; (**L**): Changes in alkaline phosphatase activity during colonic fermentation. Error bars represent the standard deviation of three independent experiments. Means with different letters are significantly different (*p* < 0.05).

Superoxide dismutase (SOD) is a class of enzymes that limit oxidative stress in the body. In addition to demonstrating efficacy in anti-tumor, anti-radiation, and anti-aging research [41], it plays a critical protective role in the gut, including maintaining barrier function, regulating immune cell balance, sustaining microbiota stability, and inhibiting pro-inflammatory signaling pathways; it may also serve as a diagnostic marker for colitis [42,43]. In the stomach and small intestine phases (Figure 3A), SOD activity was highest in the cell-wall-broken pine pollen (29 U/mL), whereas the cell-wall-broken extract lost almost all of its activity after digestion in the small intestine (<2 U/mL). During the colonic fermentation phase, significant gender differences in SOD activity were observed in the PB group (Figure 3B). In males, SOD activity reached as high as 9.5 U/mL at 4 h but declined rapidly thereafter, whereas in females, it started at only 3.7 U/mL and dropped to 0.6 U/mL by 24 h, suggesting that their gut microbiota exhibited short-term activity but lacked the ability to sustain it. The SOD values in the T group and the WPB group remained consistently below 2 U/mL, indicating weak ability to induce microbial activity. Overall, broken pine pollen most effectively activated the overall fermentative metabolism of the colonic microbiota, driving the continuous secretion and accumulation of apparent total SOD catalytic activity in the fermentation broth. In contrast, the unbroken pine pollen and extract groups exhibited very limited driving effects on microbiota-related enzymatic metabolism.

α-amylase (AMY) plays a key role in the digestion of carbohydrates in living organisms and in the utilization of starch as a carbon source by microorganisms [44]. AMY activity was significantly higher in the small intestine than in the stomach (Figure 3C), and the cell-broken extract exhibited the highest enzymatic activity in both digestive stages, indicating its potential advantage in the initial stages of carbohydrate hydrolysis. During the colonic fermentation phase (Figure 3D), levels in the PB group were nearly zero for males, while females reached a peak of 0.727 U/mL during the first 8 h, which was the highest among all groups. However, the activity subsequently declined rapidly, indicating that the microbial community is capable of explosive growth but lacks sustainability. There was no significant difference in activity between men and women in Group T, with levels remaining stable overall between 0.1 and 0.2. In the WPB group, activity persisted throughout the entire cycle, peaking at 0.186. In females, activity peaked within the first 8 h but then dropped sharply to near-zero levels. In summary, middle-aged females exhibited lower α-amylase activity. Previous studies have shown that the human gut microbiota begins to undergo significant structural changes as early as middle age, with a marked reduction in the relative abundance of core beneficial bacteria such as Bifidobacterium being a central feature of this transitional period. Clinically, this often leads to a stage-wise decline in the glycolytic capacity for complex plant polysaccharides and the production of short-chain fatty acids in middle-aged populations [45]. This may explain the low α-amylase activity observed in the colon of middle-aged individuals.

Lipases (LPSs) are essential enzymes in basic metabolic pathways, catalyzing esterification, transesterification, acidolysis, alcoholysis, and amideolysis reactions [46]. In living organisms, LPS plays a crucial role in the digestion, absorption, storage, and metabolism of fats. As shown in Figure 3E, the PB group exhibited the highest activity in the gastric phase (11.075 U/g), indicating that cell wall disruption significantly promotes the early release of lipase; however, activity dropped sharply in the small intestine phase, suggesting that enzymatic activity is primarily concentrated in the gastric digestion phase. In contrast, the T group showed higher activity in the small intestine phase (7.940 U/g) than in the gastric phase, which may be related to the fact that the fat-soluble components in the extract are more easily activated in the small intestine environment. During the colonic fermentation phase (Figure 3F), enzyme activity in the PB and T groups dropped to zero after 8 h of fermentation, whereas in the WPB group, it did not drop to zero until after 20 h of fermentation; moreover, values were higher in males than in females. This may be because the cell wall disruption in the PB and T groups broke down the cell wall structure of the pine pollen, causing the lipases enclosed within the cells to be released in large quantities during the gastric and small intestinal phases, whereas the intact cell wall structure of the undisturbed pine pollen allowed the lipases to continue to be released slowly and steadily during the colonic fermentation phase.

Phytoflavonoids are a class of polyphenolic secondary metabolites widely found in plants. The consumption of flavonoids offers numerous health benefits; for example, quercetin has the ability to regulate hepatic lipid metabolism, oxidative stress, and inflammation, and can alleviate metabolic-related fatty liver disease [47], while anthocyanins can improve vascular endothelial function and lower blood pressure [48]. In the stomach and small intestine stages (Figure 3G), both cell-wall-broken pine pollen and the cell-wall-broken extract significantly promoted the release of flavonoids; the highest release was observed for cell-wall-broken pine pollen in the small intestine stage (1.51 mg/g), while the cell-wall-broken extract showed the highest release in the stomach stage (1.124 mg/g). During the colonic fermentation phase (Figure 3H), the PB group exhibited the lowest flavonoid release efficiency; in males, flavonoid levels dropped to 0 between 16 and 20 h of fermentation, but rose abnormally to 0.522 mg/g at 24 h. This phenomenon requires further validation in subsequent studies and may reveal the mechanisms underlying the late-stage interactions between the microbiota and flavonoid complexes. Group T exhibited the strongest ability to release flavonoids, but there were significant gender differences: values in males were far higher than those in females, and males displayed a complex metabolic pattern with two peaks. The WPB group provided the smoothest and most sustained release; women reached a peak of 0.605 mg/g at 16 h, demonstrating the sustained-release protective effect of the cell wall.

Catalase (CAT) plays a role in preventing cellular damage and maintaining cellular integrity. Many health conditions, including diabetes, vitiligo, cardiovascular disease, Wilson’s disease, hypertension, anemia, certain skin disorders, Alzheimer’s disease, bipolar disorder, and schizophrenia, have been linked to a deficiency or dysfunction of catalase [49]. In the stomach and small intestine stages (Figure 3I), the highest catalase activity was observed in uncracked pine pollen and cracked extract, at 17.159 and 11.9979 μmol/g, respectively. The results for the colonic fermentation phase (Figure 3J) show that middle-aged women in the PB group exhibited particularly high levels (reaching 3.983 μmol/g at 4 h), indicating that cell wall disruption facilitates rapid recognition by their gut microbiota and induces CAT expression. Group T provides the most stable, gender-neutral sustained-release formulation. The WPB group exhibited the strongest activity-preserving effect on CAT, particularly in the female group, where activity peaked in the later stages (24 h activity: 3.54 μmol/g), reflecting the sustained-release effect of the cell wall.

Alkaline phosphatase (ALP) is an enzyme that catalyzes the hydrolysis of phosphates. It plays a key role in a variety of biological processes and is essential for cell division, growth, metabolism, and apoptosis [50]. Compared with normal cells, diseased cells exhibit overexpression of ALP; timely detection of ALP in serum is of great significance for the early diagnosis and monitoring of conditions such as bone diseases, hepatobiliary diseases, thyroid disorders, and cancer [51,52]. As shown in Figure 3K, ALP levels are extremely low in the stomach and small intestine (<0.03 U/mL). The apparent ALP activity detected during the subsequent colonic fermentation stage did not originate from residual plant enzymes, but rather reflected the microecological manifestation of the metabolic activity of the gut microbial community within the fermentation system. During colonic fermentation (Figure 3L), pine pollen processed by different methods differentially influenced the metabolic activity of relevant products and enzyme-producing genera in the colon by altering the spatial physical properties and apparent bioaccessibility of the substrates. During colonic fermentation, the PB group exhibited the highest ALP activity, which persisted for 24 h; however, the values for females were significantly lower than those for males. ALP activity in Group T was extremely low (<0.23 U/g), suggesting that its composition or structure may not be sufficient to activate the ALP expression pathway. The WPB group showed moderate activity, reflecting the slow-release protective effect of the cell wall; however, levels in women dropped sharply after 12 h. The overall results indicate that the structure of broken pine pollen provides the most suitable substrate bioaccessibility for the dynamic colonic microbiota to support metabolically active accumulation of total apparent ALP activity, with a significant influence of sex factors. This may be attributed to the distinctly different sex hormone profiles between males and females, which chronically shape markedly different physicochemical environments of the intestinal mucosa in vivo.

Based on the combined results of the six enzyme assays and the analysis of active components, the three methods of pine pollen processing exhibited distinct metabolic profiles during the stages of gastrointestinal digestion and colonic fermentation. Group PB exhibited a burst-like release pattern, showing the highest levels of SOD, LPS, and plant flavonoid release in the stomach and small intestine, and inducing the highest ALP activity during colonic fermentation. However, its CAT activity declined most rapidly (returning to zero within 12 h in males), and it exhibited the lowest efficiency of flavonoid release in the colon. The WPB group achieved a sustained-release effect due to its intact cell wall structure; LPS activity persisted for up to 20 h of fermentation. Notably, the CAT activity in the female group remained high at 3.54 μmol/g even after 24 h of fermentation, while the release of plant flavonoids was the most stable, demonstrating significant potential for long-term intestinal protection. Group T exhibited stable metabolic characteristics, with the highest capacity for releasing plant flavonoids (peak value of approximately 1.0 mg/g in males). AMY activity was highest during the digestive phase, while CAT activity showed a stable 24 h decline in colonic fermentation with no gender differences. However, SOD and ALP activities were extremely low, making it nearly impossible to activate the relevant microbial metabolic pathways. In addition, gender differences were observed across the board. Men performed better in the SOD, ALP, and T-group flavonoid release tests, while women showed superior performance in the CAT and LPS sustained-release tests in the WPB group. The horizontal differences in total enzyme activity observed here reflect the possibility that the metabolic activity of specific microbial communities within the fermentation system may be externally driven by substrates with different physical structures.

### 3.4. Changes in Gas Concentrations During the Fermentation of Pine Pollen in the Colon

**Figure 4:**
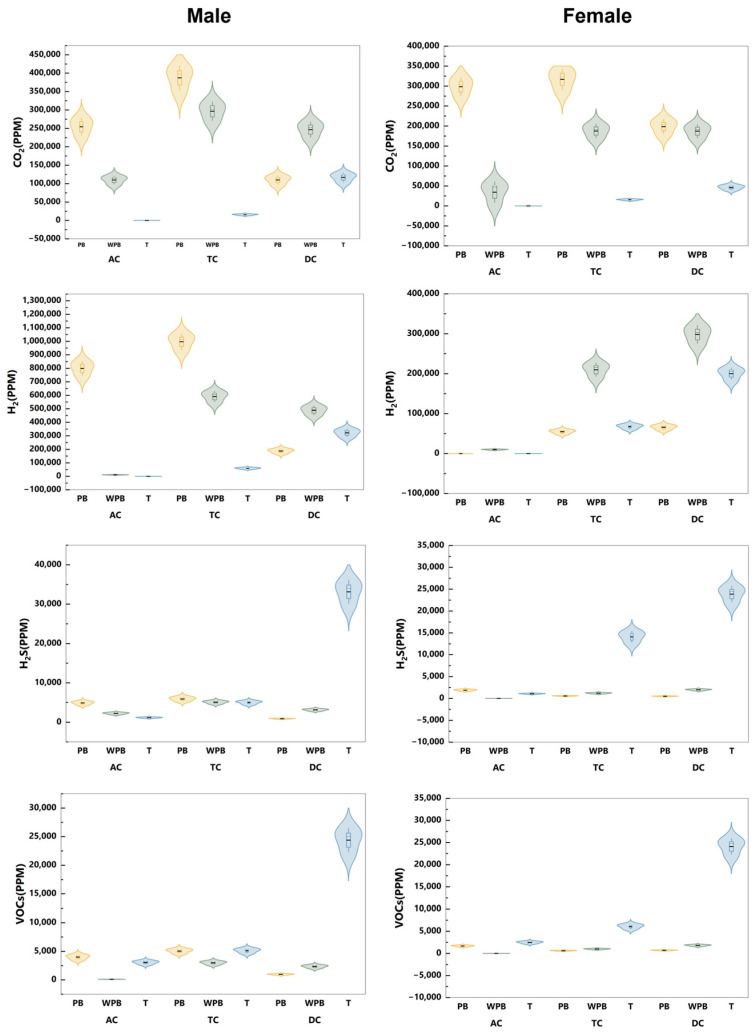
Changes in gas concentrations during colonic fermentation of the three samples.

The interaction between gas metabolism and gut function is a complex and multifaceted field of research that highlights the critical role of the gut microbiota in health and disease [53]. This study evaluated the differential utilization of pine pollen processed by different methods by the gut microbiota by measuring the concentrations of key gaseous metabolites—including H_2_, CO_2_, H_2_S, and volatile organic compounds (VOCs)—generated in an in vitro gastrointestinal digestion model. As shown in Figure 4, H_2_ and CO_2_ are key fermentation products, and their concentrations indicate substrate conversion efficiency and microbial community activity [54]. Horizontal comparison shows that the apparent concentrations of H_2_ and CO_2_ produced in the PB group were substantially higher than those in the WPB and T groups. This significant metabolic difference indicates that mechanical wall disruption removes the physical barrier, greatly enhancing the bioaccessibility and microbial utilization rate of complex polysaccharides from pine pollen in the in vitro fermentation system. However, this explosive gas production over a short period has dual physiological effects in vivo. While it indicates efficient substrate conversion, it may also imply a potential clinical risk of intestinal bloating or gastrointestinal intolerance in the host due to the escape of large amounts of substrate to the distal colon. H_2_S can be produced by the gut microbiota through the fermentation of sulfur-containing amino acids or the reduction in inorganic sulfates [55], while VOCs are generated by the fermentation of polysaccharides, proteins, and lipids by the microbiota. Generally, low concentrations of H_2_S have a beneficial effect on certain conditions, such as vasodilation and inflammation control [56]. However, when H_2_S or certain VOCs accumulate in excessive amounts in the gut over an extended period, they may have adverse effects on the intestinal barrier and host health [57,58,59]. As shown in the figure, regarding the spatial fermentation characteristics, all groups performed similarly in the ascending colon segment and remained at a low baseline (<5000 ppm). However, upon entering the distal intestinal segment, the cumulative H_2_S concentration in the T group surged to approximately 35,000 ppm and 25,000 ppm in the descending colon segment. Compared with the high-safety microecological baseline exhibited by the PB and WPB groups, the high-load sulfur-metabolism bias of the T group in the distal colon potentially carries negative pressures in clinical in vivo applications, such as host intestinal bloating, abdominal pain, and local osmotic intolerance of the colonic epithelium due to excessive gas accumulation.

In summary, the PB group exhibited the highest and most sustained carbohydrate hydrolysis efficiency, as well as the macroscopic accumulation characteristics of H_2_ and CO_2_ gases during in vitro fermentation. Driven by its compositional characteristics, the in vitro degradation and utilization of the T group tended toward a sulfur-type fermentation metabolism in the later stages. Although this characteristic exhibited a strong targeted response in vitro, caution is warranted regarding its potential negative pressure on the intestinal barrier upon long-term excessive accumulation in vivo. In contrast, the WPB group displayed a low but persistent kinetic profile. Throughout the colonic fermentation process, its cumulative gas production remained at moderate and stable levels. This low and stable gas metabolism pattern is mainly attributed to the natural sustained-release barrier effect conferred by its intact sporopollenin cell wall structure. These gas metabolism characteristics provide important in vitro behavioral clues and parametric bases for future in vivo precision intervention studies targeting different gastrointestinal tolerance models or those requiring sustained-release nutritional kinetic support.

### 3.5. Changes in the Gut Microbiota During the Fermentation of Pine Pollen in the Colon

**Figure 5:**
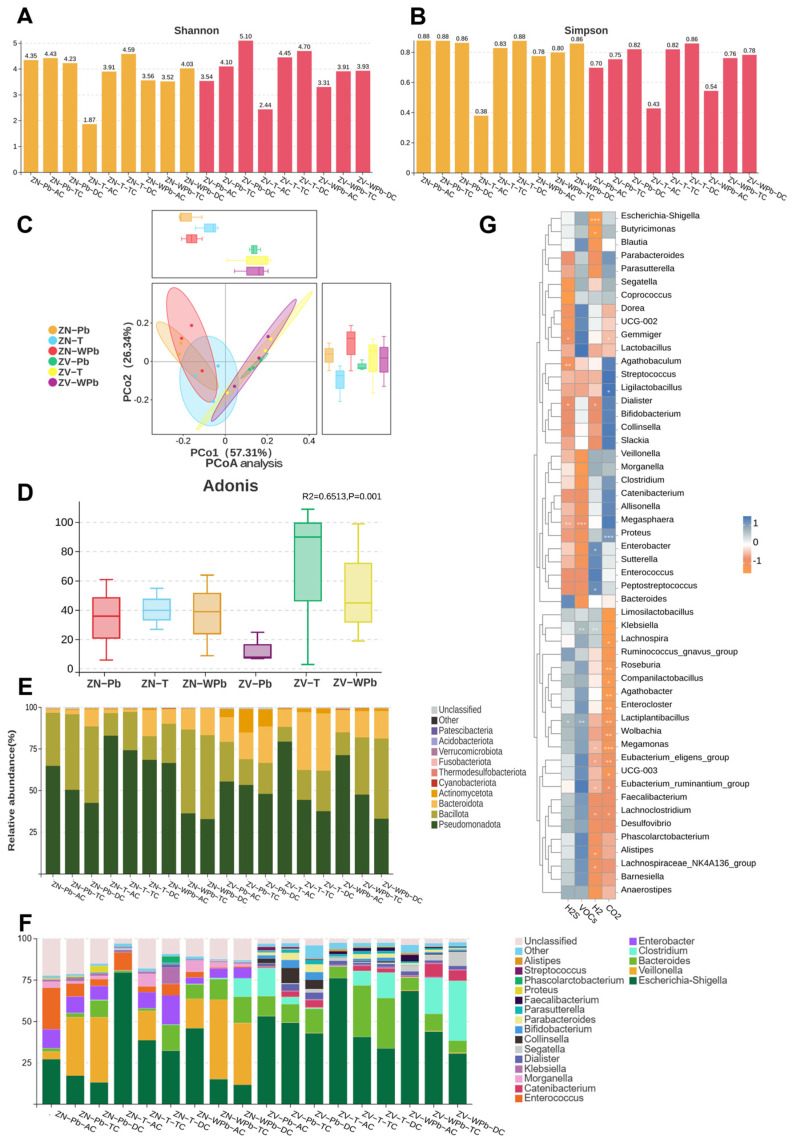
Effects of the three samples on the gut microbiota. (**A**): Shannon index; (**B**): Simpson index; (**C**): PCoA analysis; (**D**): Adonis test; (**E**): Species composition analysis at the phylum level; (**F**): Species composition analysis at the genus level; (**G**): Correlation analysis between gas and gut microbiota. Significance levels: * *p* < 0.05, ** *p* < 0.01, *** *p* < 0.001.

Alpha diversity analysis using the Shannon and Simpson indices can simultaneously reflect both the number of microorganisms in a community (richness) and the presence of any dominant high-abundance species (evenness). An analysis of alpha diversity in microbial communities across different genders and intestinal regions revealed that the Shannon index and Simpson index showed a high degree of consistency (Figure 5A,B). All three types of pine pollen caused microbial diversity and evenness to increase from the ascending colon to the descending colon, with the descending colon (DC) segment exhibiting the highest species richness and community evenness. In addition, there are significant differences in the impact of different treatment methods on diversity. Pine pollen extract (T) was associated with lower diversity in the ascending colon, suggesting that this population is more sensitive to single components, which may lead to a reduction in microbial species. In contrast, broken-wall pine pollen (PB) induced the highest levels of species richness in the descending colon of females (ZV-Pb-DC). In contrast, whole-pollen (WPB) exhibited better ecological stability in both sexes.

Beta diversity is used to measure differences in species diversity among communities across time and space. PCoA analysis was performed using the Weighted Unifrac distance algorithm (Figure 5C), combined with an Adonis (permanova) test (Figure 5D). We found that differences in community structure among samples from different treatment groups exhibited a clear gender-dependent pattern. Among middle-aged men (ZN), the effects of the six sample types on microbial community structure were relatively balanced. The effects of ZN-Pb, ZN-WPb, and ZN-T were relatively limited, primarily manifesting as maintenance-oriented regulation. In contrast, among middle-aged women (ZV), the sample’s regulatory effect on the gut microbiota was more pronounced. In particular, unbroken-wall pine pollen (ZV-WPb) and pine pollen wall extract (ZV-T) exhibited the highest levels of β-diversity, with community differences far greater than those in other groups, suggesting that they exert a stronger driving influence on the gut microbiota structure of middle-aged women.

At the phylum level, the composition of the gut microbiota in all groups underwent some degree of change (Figure 5E). In terms of gender, the proportion of dominant bacterial phyla (*Pseudomonadota* and *Bacillota*) in the male group (ZN) remained relatively stable across Pb, T, and WPB. This suggests that pine pollen supplementation did not cause any dramatic shifts in the gut microbiota of men. In the female group (ZV)—particularly in the ZV-T and ZV-WPb subgroups—the proportion of *Bacteroidota* and *Bacillota* increased significantly, while the proportion of *Pseudomonadota* decreased markedly. This is consistent with the observed changes in β-diversity. *Bacteroidota* possess polysaccharide-degrading capabilities; it is possible that the complex resistant polysaccharides (such as spore powder derivatives) abundant in pine pollen walls and extracts are efficiently utilized in the female gut, thereby inducing the specific proliferation of *Bacteroidota*.

At the genus level (Figure 5F), following intervention with the three samples, harmful bacteria in the gut microbiota decreased while beneficial bacteria increased. *Escherichia-Shigella* is positively associated with various diseases [60,61,62]. *Bifidobacterium*, a widely distributed genus of commensal bacteria, possesses beneficial homeostatic and anti-inflammatory immunomodulatory properties [63]. *Veillonella* can convert lactic acid into propionic acid [64]. *Clostridium* can anaerobically degrade uric acid into lactic acid and short-chain fatty acids (such as acetic acid and butyric acid) [65], and can effectively alleviate inflammation and allergic diseases [66]. *Bacteroides* is an abundant gut commensal bacterium that plays a crucial role in maintaining a healthy human gastrointestinal tract. It produces short-chain fatty acids such as succinic acid, acetate, butyrate, and propionate [67], and shows great promise in the treatment of fatty liver disease [68]. The relative abundance of all *Escherichia-Shigella* groups decreased significantly. Following Pb treatment, the abundance of *Bifidobacterium* in middle-aged women increased significantly, consistent with the overall trend of increased *Actinobacteria* abundance, suggesting that Pb’s beneficial effects on probiotics may be more pronounced in women. In middle-aged men, the primary effect was an increase in *Veillonella* abundance, indicating that these bacteria primarily promote lactate metabolism by producing short-chain fatty acids and improve energy metabolism and anti-inflammatory capacity by elevating propionate levels. Gender differences may be related to higher lactate production or greater sensitivity to propionate signaling in the baseline gut microbiota of men. Following T treatment, an increase in *Bacteroides* abundance was observed. The *Bacteroides* genus is a key group of bacteria involved in the breakdown of dietary fiber and the production of short-chain fatty acids. Treatment with T increases its abundance, which helps boost the production of succinic acid, acetic acid, butyric acid, and propionic acid in the gut; these metabolites can improve gut health. Following WPB treatment, the abundance of *Clostridium* increased. Due to the absence of a blank fermentation control without any pine pollen supplementation, the observed changes in microbial community structure cannot be simply attributed to the absolute prebiotic efficacy of pine pollen. However, the highly significant differences among the three experimental groups under identical basal medium formulations effectively demonstrate that the integrity of the sporopollenin exine leads to distinctly different successional tendencies in gut microbiota utilization of the substrate. For example, the PB group, with fully exposed internal contents, triggered a more rapid metabolic response from the microbiota, whereas the WPB group exhibited a more prolonged and differentiated regulatory spectrum.

Spearman’s correlation analysis identified associations between gas production and microbial genera at the genus level (Figure 5G). The heatmap reveals that members of the *Pseudomonadota* phylum, such as *Proteus* and *Enterobacter*, show a significant positive correlation with H_2_ and CO_2_, indicating that these bacterial communities are the primary contributors to gas production during the early stages of substrate degradation. However, many classic butyrate-producing bacteria, such as *Faecalibacterium* [69], *Roseburia* [70], and *Anaerostipes* [71], show a negative correlation with H_2_. This phenomenon may be due to the fact that when butyric acid-related pathways are enhanced, the H_2_ produced is rapidly consumed, leading to a decrease in net gas accumulation. *Escherichia-Shigella* shows a negative correlation with H_2_S, suggesting that it is not a major contributor to sulfur production. The sulfate-reducing bacterium *Desulfovibrio* [72] and the acetate-producing bacteria *Alistipes* showed a significant positive correlation with H_2_S. *Bacteroides* and *Lactobacillus* showed a positive correlation with VOCs. This further supports the clinical hypothesis that VOCs in intestinal gas can be used to assess intestinal barrier function and metabolic burden [73].

## 4. Conclusions

Based on a spatiotemporal dynamic evaluation system for biomimetic gastrointestinal reactors, this study systematically elucidated the differential effects of three processing methods—cell-broken, cell-intact, and cell-broken extracts—of pine pollen during simulated digestion and colonic fermentation in middle-aged individuals. The results indicate that cell wall disruption is a key structural factor determining the apparent bioaccessibility of pine pollen. In the stomach and small intestine, the cell walls of broken-wall pine pollen undergo significant disintegration, exposing their contents. This results in a rapid release of glucose, superoxide dismutase, and lipase. During fermentation in the colon, it induces the highest levels of alkaline phosphatase activity and gas production (H_2_ and CO_2_), and specifically promotes the proliferation of Bifidobacterium in middle-aged women. Unbroken-wall pine pollen achieves sustained, gradual release of nutrients through the physical barrier effect of the spore wall. Its lipase and catalase activities decline most slowly in the colon, and gas metabolism remains stable, making it suitable for long-term maintenance in individuals with sensitive digestive systems. Although pine pollen cell-wall-broken extract exhibits outstanding glucose and flavonoid release capabilities in the small intestine, its fermentation in the colon is accompanied by a specific increase in H_2_S and VOCs in the descending colon, and its ability to induce SOD and alkaline phosphatase is weak, suggesting that its metabolic characteristics are significantly spatially limited and potentially ambivalent. In addition, the effects of pine pollen on gut microbiota regulation showed significant gender differences: the microbiota structure of middle-aged women was more responsive to interventions involving both whole pollen and extracts, with a substantial increase in *Bacteroidetes* abundance, whereas the microbiota of middle-aged men remained relatively stable, with an increase in Veillonella abundance being the primary response. Due to the absence of baseline data from a blank control in the experimental design, this study does not draw conclusions regarding the absolute prebiotic efficacy of pine pollen. However, the significant lateral differences in gas production profiles, enzyme activity kinetics, and relative abundances of specific microbial genera among the PB, WPB, and T groups clearly reveal the differentially regulated patterns of pine pollen processed by different methods along the digestive tract. Specifically, wall-broken treatment (PB), by eliminating the physical barrier of the sporopollenin exine, drove an explosive release and highly efficient metabolism of nutrients and active components in the upper digestive tract. In contrast, unbroken pine pollen with an intact outer wall (WPB) tended to serve as a natural sustained-release vehicle, retaining its core nutritional matrix until reaching the distal colon, thereby inducing a more stable and prolonged microbial community response. This provides important scientific evidence and design strategies for the future development of customized pine pollen functional foods tailored to populations with different physiological needs.

## Data Availability

The original contributions presented in the study are included in the article; further inquiries can be directed to the corresponding authors.
